# Effectiveness of Digital Health Interventions in Promoting Physical Activity Among College Students: Systematic Review and Meta-Analysis

**DOI:** 10.2196/51714

**Published:** 2024-11-20

**Authors:** Siyuan Bi, Junfeng Yuan, Yanling Wang, Wenxin Zhang, Luqin Zhang, Yongjuan Zhang, Rui Zhu, Lin Luo

**Affiliations:** 1 School of Physical Education Guizhou Normal University Guiyang China

**Keywords:** digital health intervention, college students, physical activity behavior, steps, light intensity physical activity, moderate to vigorous physical activity, sedentary behavior, knowledge synthesis, systematic review, meta-analysis, physical activity, eHealth, digital health, mobile phone

## Abstract

**Background:**

Recent studies offer conflicting conclusions about the effectiveness of digital health interventions in changing physical activity behaviors. In addition, research focusing on digital health interventions for college students remains relatively scarce.

**Objective:**

This study aims to examine the impact of digital health interventions on physical activity behaviors among college students, using objective measures as outcome indicators.

**Methods:**

In accordance with the 2020 PRISMA (Preferred Reporting Items for Systematic Reviews and Meta-Analyses) guidelines, a comprehensive literature search was conducted across several databases, including MEDLINE (PubMed), Web of Science, Cochrane Library, and EBSCO (CINAHL Plus with full text), to identify relevant intervention studies published up to June 6, 2023. The inclusion criteria specified studies that examined the quantitative relationships between digital health interventions and physical activity among adults aged 18 years to 29 years, focusing on light physical activity (LPA), moderate to vigorous physical activity (MVPA), sedentary time (ST), or steps. Non–randomized controlled trials were excluded. The quality of the studies was assessed using the Cochrane Risk of Bias tool. Results were synthesized both narratively and quantitatively, where applicable. When sufficient homogeneity was found among studies, a random-effects model was used for meta-analysis to account for variability.

**Results:**

In total, 8 studies, encompassing 569 participants, were included in the analysis. The primary outcomes measured were LPA, MVPA, ST, and steps. Among these studies, 3 reported on LPA, 5 on MVPA, 5 on ST, and 3 on steps. The meta-analysis revealed a significant increase in steps for the intervention group compared with the control group (standardized mean difference [SMD] 0.64, 95% CI 0.37-0.92; *P*<.001). However, no significant differences were observed between the intervention and control groups regarding LPA (SMD –0.08, 95% CI –0.32 to 0.16; *P*=.51), MVPA (SMD 0.02, 95% CI –0.19 to 0.22; *P*=.88), and ST (SMD 0.03, 95% CI –0.18 to 0.24; *P*=.78).

**Conclusions:**

Digital health interventions are effective in increasing steps among college students; however, their effects on LPA, MVPA, and sedentary behavior are limited.

**Trial Registration:**

PROSPERO CRD42024533180; https://www.crd.york.ac.uk/prospero/display_record.php?RecordID=533180

## Introduction

### Background

The college years represent a critical phase [1] that significantly influences the levels of physical activity and the formation of healthy habits [2-4]. However, research indicates that physical activity levels among college students are generally low and rarely meet the recommended health guidelines [5]. Physical activity and sedentary behavior are interrelated behaviors that affect health and well-being and are risk factors for noncommunicable diseases. The increasing number of sedentary adults negatively impacts the prevalence of noncommunicable diseases and the overall health status of the global population [6-9]. Physical activity is a crucial protective factor that can prevent and manage major noncommunicable diseases such as cardiovascular diseases, stroke, diabetes, breast cancer, and colon cancer. Furthermore, physical activity helps prevent other significant risk factors for noncommunicable diseases, such as hypertension, overweight, and obesity, while also improving mental health, delaying the onset of dementia, and enhancing the quality of life and well-being. Based on this, the World Health Organization has developed global recommendations for promoting health through physical activity, suggesting that adults should engage in at least 150 minutes of moderate-intensity physical activity or 75 minutes of vigorous-intensity physical activity per week. However, currently, a quarter of adults worldwide fail to meet the global physical activity recommendations as set by the World Health Organization [10]. Thus, efforts to increase physical activity and reduce sedentary behavior have been identified as international public health priorities [11-13].

The lack of physical activity negatively affects the health of college students, potentially leading to psychological health issues, academic performance decline, and limited social life [14-16]. To improve college students’ physical activity behavior, digital health interventions have been recognized as a promising strategy [17,18]. Digital health is a health strategy that uses digital, mobile, and wireless technologies to support the achievement of health goals, including mobile health (mHealth) and eHealth. These technologies encompass mobile phones, tablets, web-based interventions, SMS, social media, fitness mobile apps, and wearable devices [19]. Digital health interventions may involve the use of mobile applications, social media platforms, online fitness programs, and other digital tools to provide personalized health advice, goal setting, and monitoring [20-23]. A characteristic feature of digital health interventions is their ease of deployment and the ability to target specific population groups [19,24]. The Global Action Plan on Physical Activity by the World Health Organization emphasizes the use of digital health interventions to promote physical activity behavior [25]. The advantages of digital health interventions lie in their reliability, real-time nature, and personalized customization, which can provide effective support for physical activity among college students [26-33]. Considering that college students are characterized as digital natives [34-36], scholars have conducted numerous digital health intervention studies targeting college students’ health behaviors. However, despite the extensive research on digital health interventions, the overall effectiveness of these interventions on college students’ physical activity behavior remains unclear.

Some studies have found that personalized intelligent SMS interventions have a positive impact on physical activity health outcomes [37,38]. Other research supports the effectiveness of smartphone applications in increasing physical activity [39-44]. However, there are also research findings that suggest digital health interventions may not effectively promote changes in physical activity behavior [45-47]. In addition, some studies have found that internet-based interventions have positive effects on physical activity, but with small effect sizes and heterogeneity [48,49]. Furthermore, other studies suggest that digital health interventions may improve specific health behaviors and outcomes, but with limited effectiveness [50,51]. There are also studies that indicate a small positive correlation between digital health engagement and physical activity, but the heterogeneity in the design, settings, intervention components, and outcomes of the studies may affect the validity of the conclusions [52-54]. In summary, while digital health interventions have the potential to improve college students’ physical activity behavior, further research is needed to validate their overall effectiveness.

This study focused on young adults aged 18-29 years, specifically college students in the early adulthood stage [1]. This demographic was chosen due to its unique developmental phase, characterized by a transition from adolescence to adulthood. Individuals in this group face numerous challenges, including academic pressure, changing living environments, and shifting social roles, all of which can contribute to decreased physical activity levels and an increased risk of obesity and related chronic diseases [2-4].

Digital health interventions, as a convenient, low-cost, and easily disseminated health promotion tool, have shown potential in promoting physical activity in recent years [19,24]. However, existing research presents mixed findings regarding the effectiveness of such interventions on college students’ physical activity. While some studies report positive impacts on physical activity indicators [37,38], others suggest limited or even insignificant effects [45-47].

To address this ambiguity, a meta-analysis was conducted to synthesize and analyze existing research findings. This rigorous approach aimed to provide more comprehensive and reliable evidence for evaluating the impact of digital health interventions on college students’ physical activity behavior. The findings contributed to filling the knowledge gaps in the current literature and offered empirical evidence to support the application of digital health interventions in promoting physical activity among this population.

### Objectives

The aim of this study is to assess the overall impact of digital health interventions on college students’ physical activity behavior through a meta-analysis. By synthesizing and summarizing existing research findings, the study seeks to obtain an accurate evaluation of the overall effectiveness of digital health interventions in the college student population. In this context, the following research questions are proposed: Do digital health interventions have a positive impact on college students’ physical activity behavior? Can digital health interventions significantly improve the level of physical activity among college students? Are there differences in the effectiveness of digital health interventions across different modes of physical activity?

By addressing these research questions, the study aims to develop a comprehensive understanding of the impact of digital health interventions on college students’ physical activity behavior. The findings are intended to provide scientific evidence to support the development of relevant policies and intervention measures.

## Methods

### Overview

This systematic review was conducted in accordance with the PRISMA (Preferred Reporting Items for Systematic Reviews and Meta-Analyses) 2020 guidelines [55] and was registered with PROSPERO (International Prospective Register of Systematic Reviews) under the identifier CRD42024533180. A rigorous and comprehensive literature search was executed using computer-assisted systematic search strategies. Initially, as specified in our registered protocol (CRD42024533180), the intention was to limit the search to the MEDLINE (PubMed) and Web of Science databases. However, to ensure a more comprehensive inclusion of relevant literature, the search strategy was subsequently expanded to incorporate additional databases, namely the Cochrane Library and EBSCO (CINAHL Plus with full text). Apart from this modification in the search strategy, the study design remained consistent with the original PROSPERO protocol.

### Search Strategy

This study aimed to assess the impact of digital health interventions on changes in physical activity behavior among college students. To ensure a comprehensive search, we searched MEDLINE (PubMed) and Web of Science, Cochrane library, and EBSCO (CINAHL Plus with full text) databases for relevant articles published up to June 6, 2023. The detailed search strategy, including specific keywords and search terms, is available in Multimedia Appendix 1.

### Selection Criteria

The selection criteria are shown in Textbox 1.

**List of inclusion and exclusion criteria.**

**Inclusion criteria**

- Population: the study population consists of college students aged 18-29 years. Participants with intellectual disabilities, significant cognitive impairments, or severe mobility impairments were excluded from this study.
- Interventions: the included interventions in the study are digital health–based interventions that have an impact on changes in physical activity behavior. These interventions include mHealth and eHealth, such as mobile phones, tablets (such as iPad), web-based interventions (such as web-based chats and teleconferences), SMS, social media, lifestyle or fitness smartphone apps, and wearable devices.
- Control group: the included control groups in the study consist of groups that do not receive any intervention, receive traditional interventions, or receive minimal interventions (such as providing only physical activity goals). If there are multiple intervention groups in the study, the control group’s data from and the data from the intervention group with the most digital health intervention features are extracted for analysis.
- Outcomes: this meta-analysis includes studies that measure physical activity or sedentary behavior objectively using devices (such as mobile phone data, accelerometers, pedometers). The mean and SD of the following indicators are extracted: light physical activity and moderate to vigorous physical activity, sedentary time, and step count.
- Study design: this meta-analysis only includes randomized controlled trials, where the experimental group uses technology-based interventions while the control group receives either traditional interventions (primarily oral or written health education materials) or no intervention.

**Exclusion criteria**

- Review article.
- Letter to the editor.
- Editorial.
- Articles in non-English languages (while acknowledging that excluding non-English articles may introduce language bias and limit the comprehensiveness of the findings, this review focuses solely on research published in English due to resource limitations, specifically regarding translation, time, and funding).
- Other study designs (retrospective studies, quasi-experimental designs, non–randomized controlled trials).
- Articles based on studies focused on research subjects other than college students (aged 18-29 years).
- Articles that did not collect or report data on sex.
- Articles without objective measurement of physical activity (eg, relying solely on self-report).
- Articles exclusively reporting on the feasibility or acceptability of technology-based interventions, or lacking findings pertaining to physical activity behavior outcomes, were excluded.
- Articles with incomplete outcome reporting, hindering reliable synthesis, were excluded from this analysis.

### Study Selection

To guarantee the accuracy and reliability of included studies, a rigorous screening process was implemented. Following the guidelines for meta-analysis, 2 independent reviewers conducted a blinded, duplicate screening of all retrieved articles to determine their eligibility for inclusion [56]. The screening process involved 2 stages. Initially, titles and abstracts were screened to exclude studies that clearly did not meet the inclusion criteria. Subsequently, full texts of the remaining articles were reviewed to confirm their eligibility. Throughout the screening process, discrepancies between reviewers were resolved through discussion and consensus.

### Data Extraction

The researchers used standardized forms specifically developed for this review to independently extract data from each included study, ensuring comprehensive acquisition of trial data. The extracted data encompassed the study country, study participants (population and sample size), study design (description of digital intervention measures, intervention and control group characteristics, and intervention duration), outcome measurements (measurement tools and timing), and key study outcomes (mean and SD of light physical activity [LPA], moderate to vigorous physical activity [MVPA], sedentary time [ST], and steps, or baseline and follow-up step count statistics). Average changes were computed when necessary, and in cases where SDs were not reported, they were calculated based on SE or 95% CI [57,58]. If the research papers did not adequately report the results, the researchers contacted the authors to obtain additional data. In instances where discrepancies in data extraction arose, the author team engaged in discussions and reexamined the original studies until a consensus was reached.

### Risk of Bias (Quality) Assessment

The researchers used the Cochrane Collaboration bias tool, which is integrated within the Review Manager software (version 5.4, The Nordic Cochrane Centre), and supplemented it with the CONSORT (Consolidated Standards of Reporting Trials) checklist to assess the quality of the included studies [59]. This tool comprises domains for selection bias, performance bias, detection bias, attrition bias, and reporting bias. Each study was independently assessed by 2 reviewers. In addition, publication bias was assessed using funnel plot analysis.

### Data Analysis

The statistical analysis for this study was performed using Review Manager and Stata (version 16; StataCorp LLC). Considering the challenges posed by various definitions of changes in physical activity behavior, this study decided to include only literature that solely used LPA, MVPA, ST, and steps as pre- and postintervention measurement indicators. Studies that did not provide these measurement data were excluded to ensure interpretability and meaningfulness of the meta-analysis. In addition, studies that did not provide sufficient information for calculating the mean and SD, or used statistical analyses (such as correlation coefficients, multivariate, and adjusted regression coefficients) inconsistent with the relevant meta-analysis, were also excluded. Furthermore, in cases where multiple intervention groups within a study exhibited similar characteristics of digital health interventions, the intervention group with the most digital health intervention features was selected for the meta-analysis.

The primary outcome measures for this meta-analysis were the mean and SD of LPA, MVPA, ST, and steps. For each intervention and control group in each study, the baseline and follow-up mean, SD, and participant count of physical activity were entered into the Review Manager software. Using these data, standardized mean differences (SMDs) between intervention and control group changes for each study were calculated as the measure of effect size. The SMD was chosen because although the included studies used the same units of measurement, the scales or measurement tools used across studies might have varied, making direct comparisons of weighted mean differences difficult. SMD, through standardization, can eliminate the influence of different scales, reflecting the differences in intervention effects more accurately and enhancing the interpretability of the results. If data were available from 3 or more studies, a meta-analysis was conducted, and an overall effect size was computed.

Due to the diversity in study populations and intervention designs, a random effects model approach was used to account for heterogeneity among studies. The *I*² statistic was used to assess the presence of heterogeneity, which describes the percentage of total variation in effect estimates due to heterogeneity [60,61]. The *I*² statistic was chosen as the preferred measure of variance since it is stable with small sample sizes. Heterogeneity was considered significant if the *P* value from the chi-square test was less than .10 and *I*² was greater than 50% [60,62,63].

The effect size of SMDs was interpreted using Cohen suggestion [64], where 0.2 represents a small effect size, 0.5 represents a medium effect, and 0.8 represents a large effect, with values below 0.2 considered trivial. In addition, sensitivity analyses for LPA, MVPA, ST, and steps were performed using Stata software (version 16.0) to further assess the stability of the study results.

## Results

### Study Selection

**Figure 1:**
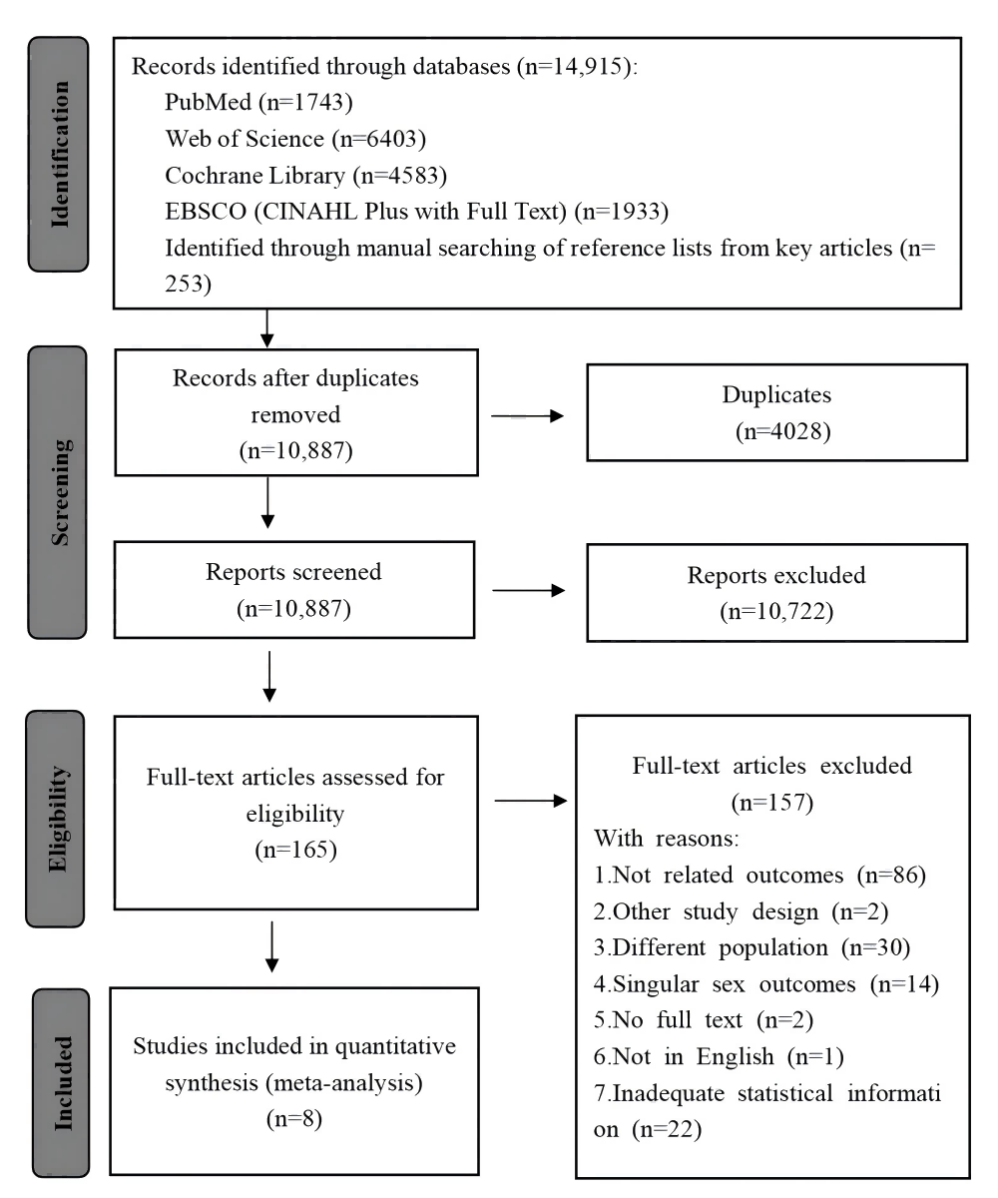
PRISMA (Preferred Reporting Items for Systematic Reviews and Meta-Analyses) diagram of the searching and screening processes.

Initially, a total of 14,915 articles were identified for evaluation across 4 databases, such as 1743 from PubMed, 6403 from Web of Science, 4583 from the Cochrane Library, and 1933 from EBSCO (CINAHL Plus with full text). In addition, 253 articles were identified through manual searching of reference lists from key articles. After excluding 4028 duplicate records from multiple databases and search channels, we proceeded to exclude 10,722 articles that were irrelevant to the scope of this study based on their titles and abstracts. From the remaining 165 articles, an additional 157 were deemed ineligible for inclusion in this meta-analysis, as they did not meet the predetermined inclusion criteria. Ultimately, a total of 8 relevant research articles were identified and included in this meta-analysis. The detailed flowchart of the literature search process is presented in Figure 1.

### Study Characteristics

#### Overview

A total of 8 studies were included in this meta-analysis, all of which were randomized controlled trials published in English between 2014 and 2023. The main characteristics of these 8 studies are provided in Multimedia Appendix 2 (PRISMA checklist provided in Multimedia Appendix 3). It was reported that four studies [45,46,50,65] were not registered on ClinicalTrials.gov, while 4 studies [47,51,66,67] were registered on the website. Among these 8 studies, 4 were conducted in Australia [45], Spain [66], China [46], and Germany [65], respectively, while the remaining 4 studies were conducted in the United States [47,50,51,67].

#### Participants

The sample sizes of these 8 studies ranged from 34 to 187 participants, with a total of 569 participants. All included studies targeted individuals between the ages of 18 and 30 years, specifically university students. Among these, except for 1 study [50] which did not report or calculate the SD of participant age, the remaining 7 studies [45-47,51,65-67] reported or calculated both the mean and SD of participant age.

The 8 studies exhibited varying gender distributions among their participants. In a study by Hebden et al [45], women comprised 80% (n=41) of the 51 participants. Similarly, a study by Kim et al [47] included 187 participants with 62% (n=116) identifying as female. The study by Miragall et al [66] comprised 52 participants, of whom 86% (n=45) were women. A study by Pope at al [51], with 38 participants, had 74% (n=28) female representation. The study by Al-Nawaiseh et al [67] included 114 participants, with women constituting 81% (n=92) of the sample. The study by Lau et al [46] had a more balanced gender distribution, with 61% (n=34) of its 56 participants being female. The study by Pope and Gao [50], involving 44 participants, had 73% (n=32) female participants. Finally, the study by Kellner et al [65], with the smallest sample size of 34, had the highest female representation at 85% (n=29).

#### Interventions

All primary interventions in the studies aimed to increase physical activity, although one study [45] aimed to evaluate the impact on body weight, and another study [50] aimed to assess the impact on health outcomes. The methods used in the intervention group included social media [46], smart text messaging [65], mobile digital applications [45,67], smart wearable technology devices (such as smartwatches and pedometers) [47,66], or a combination of social media and smartphone apps [50], or a combination of social media and wearable technology devices [51]. The control group either did not receive any intervention [46,65] or only received traditional/standard interventions [45,47,50,51,66,67]. The duration of the interventions varied from 3 weeks [66] to 4 weeks [46], 5 weeks [65], 10 weeks [50], 12 weeks [45,51,67], and 15 weeks [47].

#### Outcomes

All included studies reported multiple objective measures of physical activity. In total, 3 studies (n=276) reported LPA, 5 studies (n=369) reported MVPA, 5 studies (n=354) reported ST, and 3 studies (n=215) reported steps.

For measuring LPA, MVPA, and ST, the 8 studies used the following tools: ActiGraph GT3X+ accelerometer [68], ActiGraph accelerometer (GT1M model) [69], SenseWear Pro Armband mobile device [70], ActiGraph Actitrainer (ActiGraph LLC) [71], ActiGraph Link accelerometer [72], and ActivPal accelerometer (Pal Technologies) [73]. The tools used to measure steps included the Pacer pedometer, Accupedo pedometer, and Fitbit One pedometer.

### Risk of Bias in Included Studies

**Figure 2:**
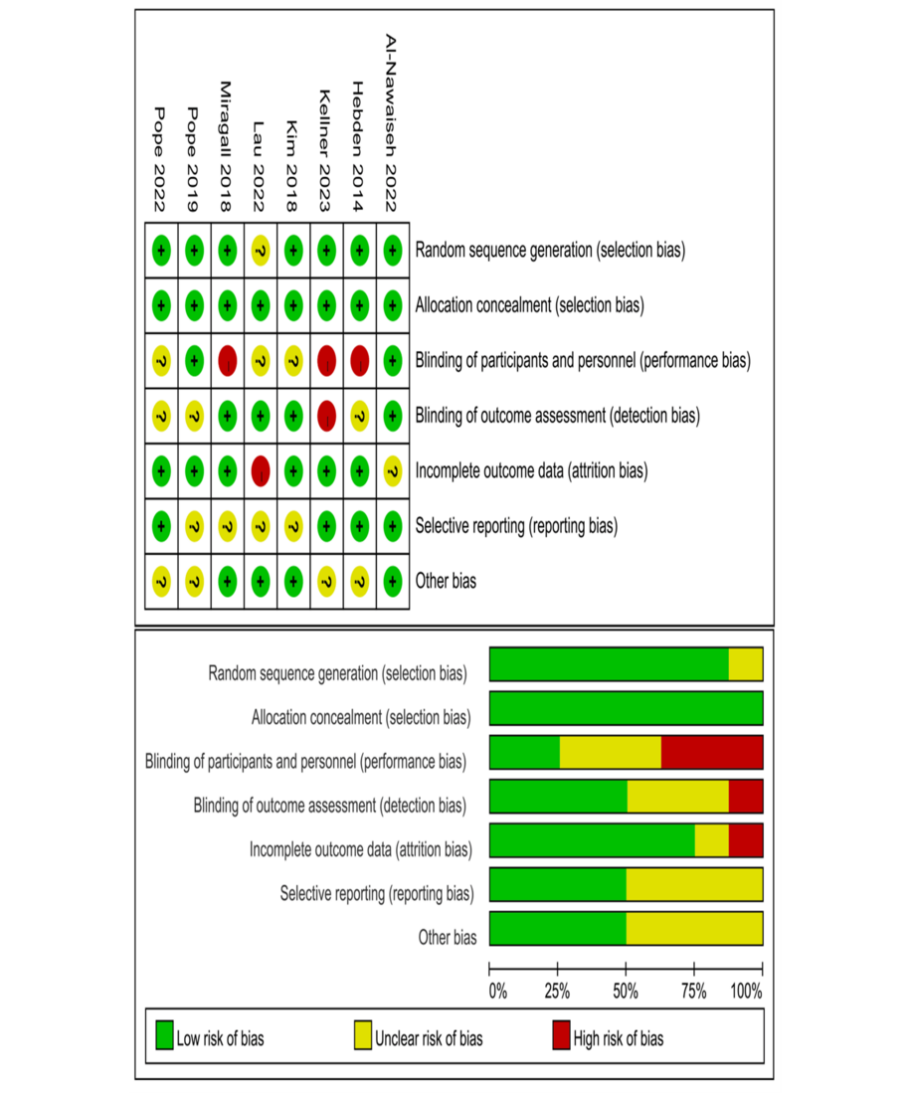
Risk of bias summary. These are authors’ judgments of each methodological quality item for each included study. Plus signs (+) indicate high methodological quality (low risk of bias); minus signs (-) indicate low methodological quality (high risk of bias); question marks (?) indicate unclear methodological quality (reported information about what happened in the study was insufficient).

The methodological rigor of the included studies was evaluated using the Cochrane Risk of Bias tool, which assesses 6 domains, namely selection bias, performance bias, detection bias, attrition bias, reporting bias, and other biases. Each domain was assessed as having a low risk, high risk, or unclear risk of bias. The overall quality of each study was then classified as high, moderate, or low based on the assessment results (Figure 2 [45-47,50,51,65-67]).

The included studies generally demonstrated moderate methodological quality, meeting average quality assessment standards. Notably, all but one study [46] explicitly described a robust random sequence generation process for participant allocation, minimizing selection bias. Furthermore, all studies reported baseline characteristics and used valid, objective measures for assessing physical activity outcomes, reducing the risk of detection bias.

However, the nature of the interventions presented challenges in achieving blinding of participants and personnel, potentially introducing performance bias. Specifically, while intention-to-treat analysis was used in all but 4 studies [46,65-67], mitigating attrition bias, the lack of blinding may have influenced participant behavior and data interpretation.

Regarding reporting bias, all studies, with the exception of Lau et al [46], provided sufficient statistical data for analysis. However, only 5 studies [46,47,50,51,67] reported between-group differences in changes over time, potentially limiting the comprehensiveness of the findings.

### Meta-Analysis

**Figure 3:**
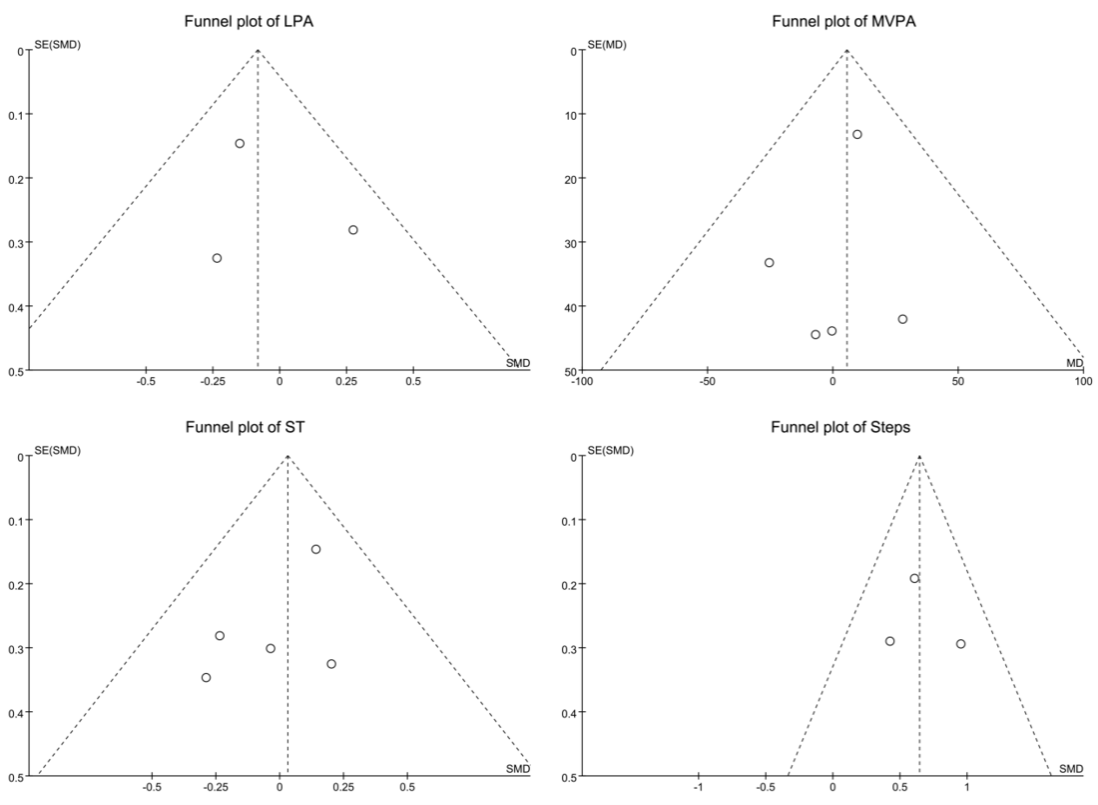
Funnel plot of light physical activity (LPA), moderate to vigorous physical activity (MVPA), sedentary time (ST), and steps.

Figure 3 displays the funnel plots for these measures, assessing publication bias and heterogeneity. The plots for LPA and steps indicate low publication bias and heterogeneity, with data points symmetrically distributed around the funnel’s center, suggesting consistent effect sizes. In contrast, the MVPA plot shows higher heterogeneity and potential publication bias, with data points more dispersed and deviating from the center line. The ST plot, while having some points at the funnel’s edges, overall indicates slight publication bias and low heterogeneity, with generally consistent effect sizes. These findings highlight the need for researchers to consider heterogeneity and potential publication bias when interpreting results for different physical activity types and sedentary time (ST).

**Figure 4:**
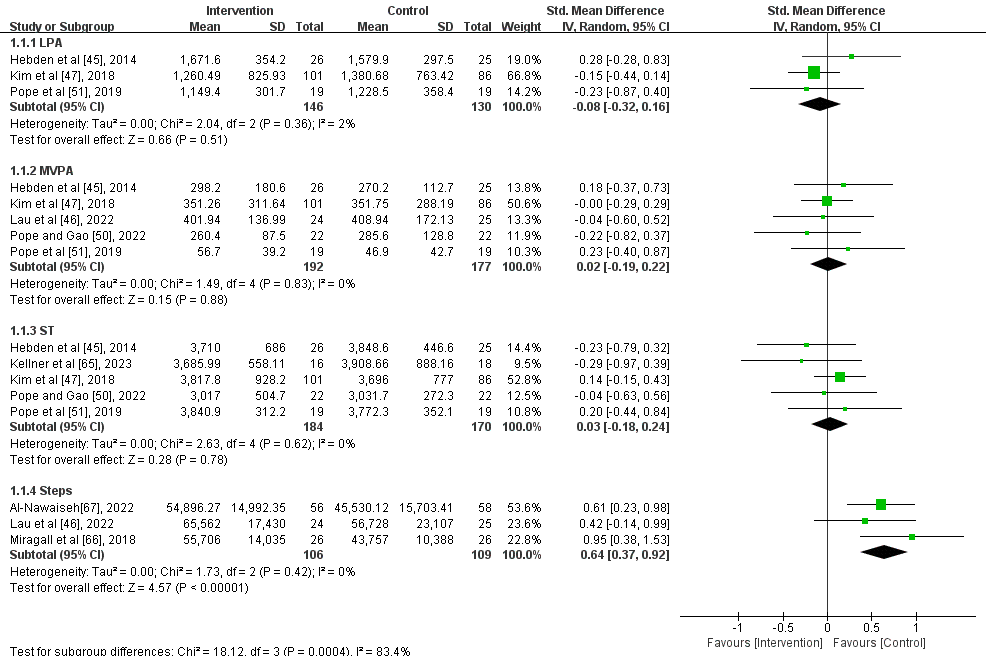
Forest plot of light physical activity (LPA), moderate to vigorous physical activity (MVPA), sedentary time (ST), and steps per week [45-47,50,51,65-67].

The results of the meta-analysis showed the forest plot (Figure 4 [45-47,50,51,65-67]) for the 4 outcome measures: LPA, MVPA, ST, and steps.

Regarding LPA, 3 studies involving 276 participants reported changes in weekly LPA. The results revealed a small SMD between the intervention and control groups, slightly favoring the control group (SMD –0.08, 95% CI –0.32 to 0.16; Figure 4 [45-47,50,51,65-67]). However, this difference was not statistically significant (*P*=.51). In addition, the results showed very low heterogeneity (*I*²=2%).

For MVPA, 5 studies involving 369 participants reported changes in weekly MVPA. The results indicated a small SMD between the intervention and control groups, slightly favoring the intervention group (SMD 0.02, 95% CI –0.19 to 0.22; Figure 4 [45-47,50,51,65-67]). However, this difference was not statistically significant (*P*=.88). Furthermore, the results showed no heterogeneity (*I*²=0%).

Regarding ST, 5 studies involving 354 participants reported changes in weekly ST. The results showed a small SMD between the intervention and control groups, slightly favoring the intervention group (SMD 0.03, 95% CI –0.18 to 0.24; Figure 4 [45-47,50,51,65-67]). However, this difference was not statistically significant (*P*=.78). In addition, the results showed no heterogeneity (*I*²=0%).

For steps, 3 studies involving 215 participants reported changes in weekly step counts. The results revealed a large SMD between the intervention and control groups, significantly favoring the intervention group (SMD 0.64, 95% CI 0.37-0.92; Figure 4 [45-47,50,51,65-67]), with a statistically significant difference (*P*<.001). Furthermore, the results showed no heterogeneity (*I*²=0%).

### Sensitivity Analysis

The research findings of the 4 outcome measures, namely LPA, MVPA, ST, and steps, indicate that the point estimates of the combined effect sizes obtained after excluding a specific study fall within the 95% CI of the overall combined effect size. This suggests that the exclusion of a particular study does not significantly alter the results, indicating a considerable stability in the evaluation of the 4 outcome measures. The sensitivity analysis charts corresponding to each outcome measure are presented in figures.

**Figure 5:**
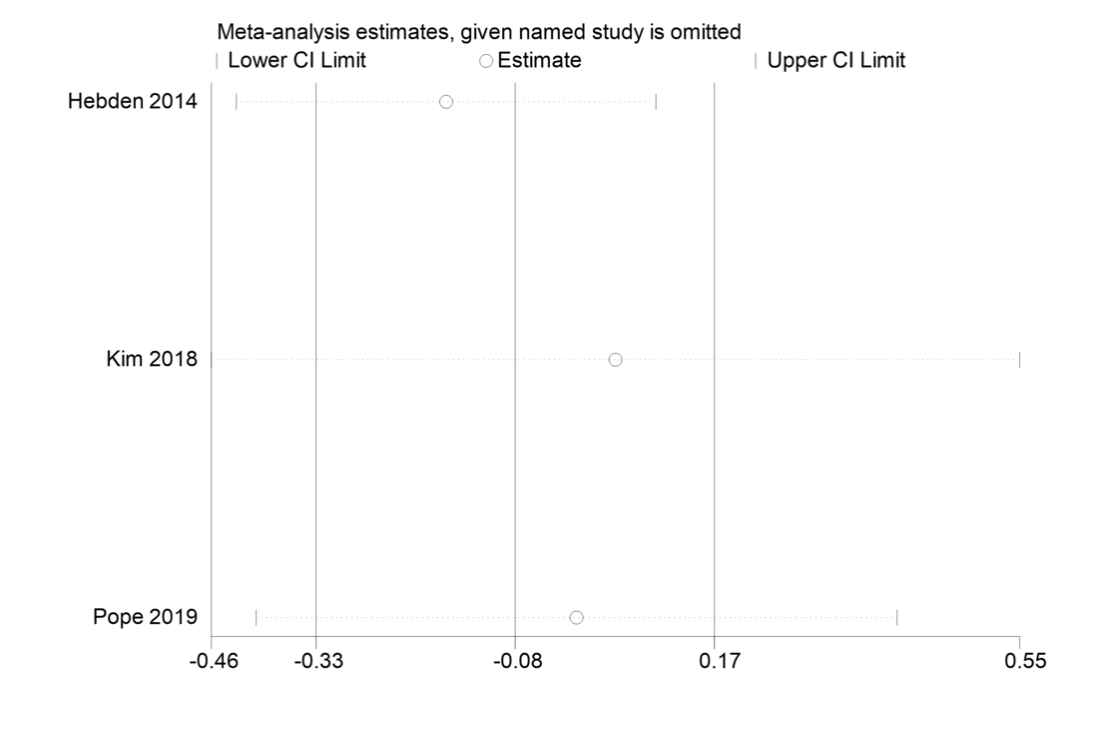
Sensitivity analysis chart of light physical activity (LPA) [45,47,51].

The sensitivity analysis chart for LPA is shown in Figure 5 [45,47,51].

The vertical line labeled “Estimate” represents the combined results of all included studies, while the lines on either side denote the upper and lower confidence intervals of these results. A circle corresponding to any particular study indicates the combined results of the remaining studies after excluding that specific study. If, upon exclusion of a study, the circle remains close to the “Estimate” line and within the original confidence interval, it suggests that the exclusion of this study does not significantly alter the combined results. This consistency implies that the meta-analysis results are robust.

**Figure 6:**
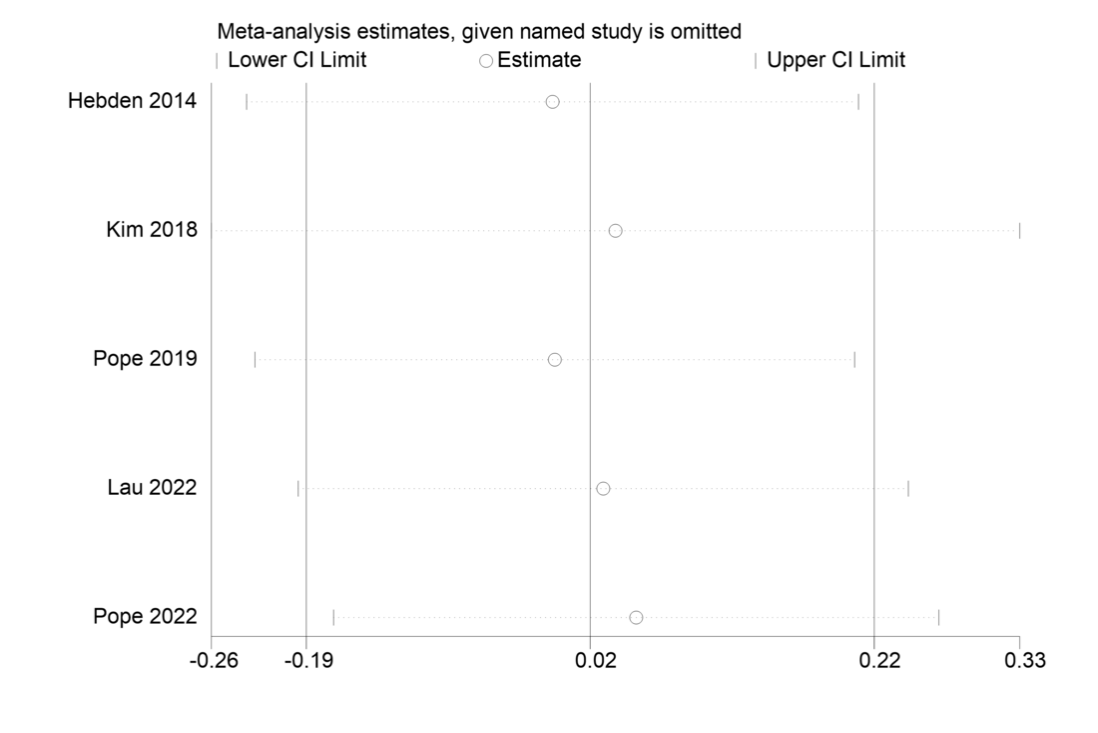
Sensitivity analysis chart of moderate to vigorous physical activity (MVPA) [45-47,50,51].

The sensitivity analysis charts for MVPA, ST, and steps are shown in Figures 6 [45-47,50,51], 7 [45,47,50,51,65], and 8 [46,66,67], respectively.

**Figure 7:**
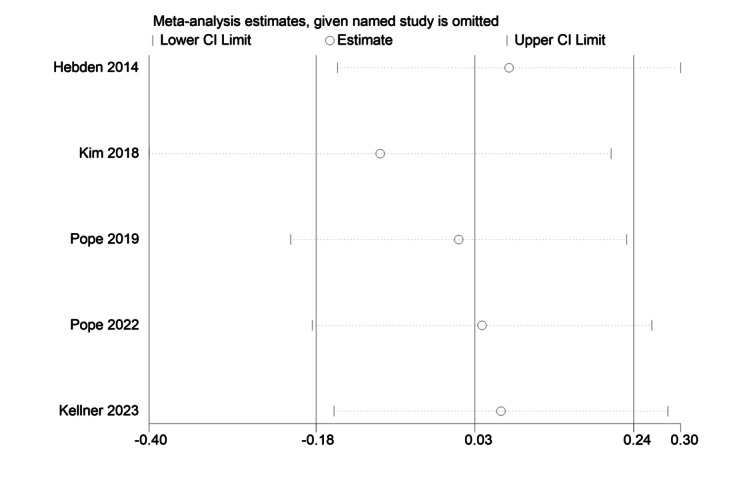
Sensitivity analysis chart of sedentary time (ST) [45,47,50,51,65].

**Figure 8:**
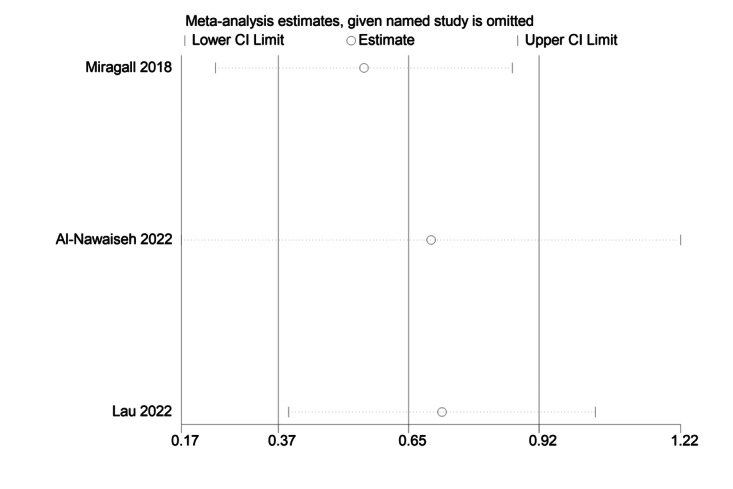
Sensitivity analysis chart of steps [46,66,67].

## Discussion

### Principal Findings

The results of the included analysis demonstrate that digital health interventions are a stable and effective strategy for significantly improving the step count of college students, thereby enhancing their physical health. This conclusion is supported by 3 randomized controlled trials included in the analysis [46,66,67]. With the widespread use of health trackers and mobile devices, daily step count has become a convenient and reliable indicator for measuring individual physical activity levels [74,75]. Research indicates that increasing daily step count has significant benefits for both physical and mental health [76]. First, increasing step count enhances cardiopulmonary function and oxygen uptake, thereby improving cardiovascular function [75,77,78]. Second, walking is a low-intensity aerobic exercise that can help lower blood pressure, control blood sugar levels, and prevent cardiovascular diseases. In addition, walking aids in calorie expenditure, weight control, and reduces the risk of obesity. Furthermore, walking helps release physical and psychological stress, improving emotional and mental well-being [79-81]. Further studies have demonstrated a correlation between daily step count and all-cause mortality. Individuals who walk 8000 to 12,000 steps per day have a lower all-cause mortality rate compared with those who walk only 4000 steps per day. This association has been validated across different age groups, genders, and ethnicities [82,83]. Therefore, focusing on step count and increasing it through intervention measures is of significant importance. Digital health interventions provide a convenient and practical approach to help college students increase their step count. By using digital health technologies such as the internet, smartphone apps, text messaging services, and wearable devices, individuals can monitor their step count, set goals, receive reminders and feedback, and more. This intervention not only alerts individuals to their walking behavior but also provides personalized advice and support to encourage increased step count.

However, the analysis results of this study indicate that digital health interventions may not be an effective method for significantly influencing the levels of low-, moderate-, and high-intensity physical activity and ST among college students. Although previous research has demonstrated that increasing the duration of low, moderate, and high-intensity physical activity can significantly reduce the negative impact of ST and improve risk factors associated with cardiovascular diseases, such as hypertension, hyperglycemia, hyperlipidemia, and mortality [84-87]. However, in this study, no significant impact of digital health interventions on the levels of low, moderate, and high-intensity physical activity and ST among college students was observed. It is worth noting that in this study, the impact of digital health interventions on step count was significant, but not significant for other modes of physical activity. This may be due to the fact that walking is a low-intensity aerobic exercise that is easily accepted and maintained by college students. In contrast, changing high-intensity physical activity and sedentary behaviors may require more personalized support and intervention measures.

Specifically, step count differs from LPA measured by accelerometers, and thus, the significance of step count does not represent the significance of LPA. Loprinzi and Cardinal [88] found that while an increase in step count is significantly associated with health, the correlation between step count and total LPA is low, indicating that the significance of step count is not equivalent to LPA. Tudor-Locke et al [89] also noted that step count primarily measures walking, whereas LPA encompasses a broader range of low-intensity activities.

In previous studies, the impact on LPA and step count yielded different results. Hebden [45] found that LPA levels significantly increased in both the intervention and control groups, with no significant difference between them. Kim et al [47] reported a significant decrease in LPA time in the intervention group, with no significant change in the control group, yet no significant difference between the groups. Pope et al [51] observed a decrease in LPA time in the intervention group and no significant change in the control group, with no significant difference between the groups. Regarding step count, Miragall et al [66] found a significant increase in daily steps in the intervention group, with no significant change in the control group, and a significant difference between the groups. Lau et al [46] found no significant difference in objectively measured step count between the intervention and control groups. Al-Nawaiseh et al [67] observed a significant increase in weekly steps from baseline to follow-up in the intervention group, with no significant change in the control group, and a significant difference between the groups.

Therefore, to effectively promote low-, moderate-, and high-intensity physical activity among college students and improve their sedentary behaviors, it is necessary to explore more effective methods and continue this investigation in future research. This may involve providing more personalized intervention measures, such as customized exercise plans, individual goal setting, and regular feedback. In addition, combining social support and motivational mechanisms, such as team activities and competitions, may further encourage college students to engage in more high-intensity physical activity and reduce sedentary behaviors.

In light of the above, digital health interventions have been identified as an effective strategy to enhance step count and improve the physical health status of college students. By monitoring step count, setting goals, and providing personalized advice and support, digital health interventions can assist college students in increasing their daily step count. However, further research is needed to investigate the effectiveness of interventions targeting other modes of physical activity and to delve into the mechanisms underlying digital health interventions. With the continuous advancement of digital health technologies, future studies should explore more precise and personalized intervention approaches and conduct in-depth investigations into the mechanisms of digital health interventions to provide a more comprehensive and profound understanding.

### Strengths and Limitations

The included studies in this meta-analysis used various intervention measures, including eHealth and mHealth technologies such as the internet, smartphone apps, SMSs, and wearable devices. All studies adhered to the standards of randomized controlled trials and used digital devices for objective measurement of physical activity outcome indicators. The research findings presented detailed results before, during, and even after follow-up, demonstrating high quality and stability. However, a limitation of this study is the relatively small sample size, which calls for further investigation.

The methodological rigor of the included studies was evaluated using the Cochrane Risk of Bias tool. The results showed that the studies generally had moderate methodological quality, meeting average quality assessment standards. Notably, except for 1 study (Lau et al [46]), all studies explicitly described a robust random sequence generation process for participant allocation, minimizing selection bias. In addition, all studies reported baseline characteristics and used valid, objective measures to assess physical activity outcomes, reducing detection bias.

However, the nature of the interventions posed challenges in achieving blinding of participants and personnel, potentially introducing performance bias. Specifically, although most studies [46,65-67] used intention-to-treat analysis to mitigate attrition bias, the lack of blinding could have influenced participant behavior and data interpretation. Regarding reporting bias, except for Lau et al [46], all studies provided sufficient statistical data for analysis. However, only 5 studies [46,47,50,51,67] reported between-group differences in changes over time, potentially limiting the comprehensiveness of the findings.

Subjective measurement of physical activity and studies involving noncollege student populations were excluded in this analysis, reducing the subjectivity and instability associated with self-report data and enhancing the reliability and consistency of the results. The accuracy of measuring LPA, MVPA, and ST may be influenced by the specific devices and wear-time protocols used in each study. This variability could result in observed inconsistencies. Sensitivity analysis further validated the stability and effectiveness of the meta-analysis results. These rigorous inclusion and exclusion criteria minimized heterogeneity and increased the credibility of the results. However, due to the limited number of included studies, subgroup analysis was not conducted, preventing an understanding of the differences in effects among different populations, intervention measures, and time points.

The key strength of this analysis lies in its focus on the impact of digital health interventions on college students, as well as the inclusion of 4 indicators of physical activity behavior. This may be the first meta-analysis on the effects of digital health interventions specifically targeting college students. However, it is important to note that this study only included English articles, which may have influenced the research sample and compromised the stability of the results. In addition, the included studies mainly originated from Western countries, lacking research on college student populations in non-Western contexts, thus potentially limiting the generalizability of the results. Further research is needed to explore the impact of digital health interventions on physical activity among college students in the future.

### Conclusions

According to the research findings, digital health interventions have shown significant effects on step counts among college students. However, the impact on LPA, moderate to high-intensity physical activity, and sedentary behavior may not be evident. It is important to note that these conclusions are based on a limited number of studies, and further research is needed to validate these findings. Specifically, more research is needed to investigate the impact of digital health interventions on increasing step counts among college students. Therefore, future studies should further explore the effects of digital health interventions on changes in physical activity behavior among college students, particularly in promoting increases in step counts. This will contribute to a better understanding of the effectiveness of digital health interventions and provide scientific evidence for the development of effective intervention strategies.

## Appendix

Multimedia Appendix 1 Searching Strategy.

Multimedia Appendix 2 Study Characteristics.

Multimedia Appendix 3 PRISMA checklist.

## Abbreviations

CONSORT: Consolidated Standards of Reporting Trials
eHealth: electronic health
LPA: light intensity physical activity
mHealth: mobile health
MVPA: moderate to vigorous physical activity
PRISMA: Preferred Reporting Items for Systematic Reviews and Meta-Analyses
PROSPERO: International Prospective Register of Systematic Reviews
SMD: standardized mean difference
ST: sedentary time
