# Supplementary material for: Effectiveness of Digital Health Interventions in Promoting Physical Activity Among College Students: Systematic Review and Meta-Analysis
Source: J Med Internet Res. 2024 Nov 20;26:e51714. doi: 10.2196/51714 (PMC11618011; doi:10.2196/51714)
Supplement: Multimedia Appendix 1 [file jmir_v26i1e51714_app1.pdf]

Multimedia Appendix 1. Searching Strategy 1

**MEDLINE(PubMed):**
(((((((((Young Adult[MeSH Terms]) OR (college student*[Title/Abstract])) OR (university student*[Title/Abstract])) OR (Adults, Young[Title/Abstract])) OR (Adult, Young[Title/Abstract])) OR (Young Adults[Title/Abstract])) OR (undergraduate[Title/Abstract])) OR (Postgraduate[Title/Abstract])) OR (Master student*[Title/Abstract])) OR (doctoral student*[Title/Abstract])) OR (Higher education student*[Title/Abstract])
((((((((((((((((((((((((((((((((((mobile health[Title/Abstract]) OR (ehealth[Title/Abstract])) OR (mhealth[Title/Abstract])) OR (mobile technology[Title/Abstract])) OR (Internet[Title/Abstract])) OR (cellular phone[Title/Abstract])) OR (cellular phones[Title/Abstract])) OR (smartphone[Title/Abstract])) OR (telecommunications[Title/Abstract])) OR (mobile applications[Title/Abstract])) OR (web-based[Title/Abstract])) OR (mobile apps[Title/Abstract])) OR (portable electronic app[Title/Abstract])) OR (portable software app[Title/Abstract])) OR (text message[Title/Abstract])) OR (SMS[Title/Abstract])) OR (short message service[Title/Abstract])) OR (portable game[Title/Abstract])) OR (computers, handheld[Title/Abstract])) OR (PDA[Title/Abstract])) OR (personal digital assistant[Title/Abstract])) OR (social media[Title/Abstract] OR social media health[Title/Abstract])) OR (Twitter[Title/Abstract])) OR (tweets[Title/Abstract])) OR (Facebook[Title/Abstract])) OR (Instagram[Title/Abstract])) OR (Wechat[Title/Abstract])) OR (Tiktok[Title/Abstract])) OR (mobile fitness apps[Title/Abstract])) OR (online social networking[Title/Abstract])) OR (virtual reality[Title/Abstract])) OR (avatars[Title/Abstract])) OR (online gaming[Title/Abstract])) OR (video games[Title/Abstract])) OR (Digital health[Title/Abstract])) OR (Telehealth[Title/Abstract])
(((((((((((((((((((Exercise[MeSH Terms]) OR (Motor activity[MeSH Terms])) OR (Sports[MeSH Terms])) OR (daily physical activit*[Title/Abstract])) OR (walk*[Title/Abstract])) OR (leisure activit*[Title/Abstract])) OR (physical fitness[Title/Abstract])) OR (health[Title/Abstract])) OR ((health behavior[Title/Abstract])) OR (weight loss[Title/Abstract])) OR (obesity[Title/Abstract])) OR (overweight[Title/Abstract])) OR (Physical activity[Title/Abstract])) OR (Fitness[Title/Abstract])) OR (Workout[Title/Abstract])) OR (Training[Title/Abstract])) OR (Athletics[Title/Abstract])) OR (Recreation[Title/Abstract])) OR (Behavior change[Title/Abstract])) OR (Health behavior change[Title/Abstract])) OR (Physical activity behavior change[Title/Abstract])
((((((clinical trial[Title/Abstract]) OR (pilot study[Title/Abstract])) OR (randomized controlled clinical trial[Title/Abstract])) OR (Randomized controlled trial[Title/Abstract])) OR (RCT[Title/Abstract])) OR (Quasi-experimental study[Title/Abstract])) OR (Intervention study[Title/Abstract])

Combined: ((((((((clinical trial[Title/Abstract]) OR (pilot study[Title/Abstract])) OR (randomized controlled clinical trial[Title/Abstract])) OR (Randomized controlled trial[Title/Abstract])) OR (RCT[Title/Abstract])) OR (Quasi-experimental study[Title/Abstract])) OR (Intervention study[Title/Abstract])) AND (((((((((((((((((((Exercise[MeSH Terms]) OR (Motor activity[MeSH Terms])) OR (Sports[MeSH Terms])) OR (daily physical activit*[Title/Abstract])) OR (walk*[Title/Abstract])) OR (leisure activit*[Title/Abstract])) OR (physical fitness[Title/Abstract])) OR (health[Title/Abstract])) OR ((health behavior[Title/Abstract])) OR (weight loss[Title/Abstract])) OR (obesity[Title/Abstract])) OR (overweight[Title/Abstract])) OR (Physical activity[Title/Abstract])) OR (Fitness[Title/Abstract])) OR (Workout[Title/Abstract])) OR (Training[Title/Abstract])) OR (Athletics[Title/Abstract])) OR (Recreation[Title/Abstract])) OR (Behavior change[Title/Abstract])) OR (Health behavior change[Title/Abstract])) OR (Physical activity behavior change[Title/Abstract]))) AND ((((((((((((((((((((((((((((((((((mobile health[Title/Abstract]) OR (ehealth[Title/Abstract])) OR (mhealth[Title/Abstract])) OR (mobile technology[Title/Abstract])) OR (Internet[Title/Abstract])) OR (cellular phone[Title/Abstract])) OR (cellular phones[Title/Abstract])) OR (smartphone[Title/Abstract])) OR (telecommunications[Title/Abstract])) OR (mobile applications[Title/Abstract])) OR (web-based[Title/Abstract])) OR (mobile apps[Title/Abstract])) OR (portable electronic app[Title/Abstract])) OR (portable software app[Title/Abstract])) OR (text message[Title/Abstract])) OR (SMS[Title/Abstract])) OR (short message service[Title/Abstract])) OR (portable game[Title/Abstract])) OR (computers, handheld[Title/Abstract])) OR (PDA[Title/Abstract])) OR (personal digital assistant[Title/Abstract])) OR (social media[Title/Abstract] OR social media health[Title/Abstract])) OR (Twitter[Title/Abstract])) OR (tweets[Title/Abstract])) OR (Facebook[Title/Abstract])) OR (Instagram[Title/Abstract])) OR (Wechat[Title/Abstract])) OR (Tiktok[Title/Abstract])) OR (mobile fitness apps[Title/Abstract])) OR (online social networking[Title/Abstract])) OR (virtual reality[Title/Abstract])) OR (avatars[Title/Abstract])) OR (online gaming[Title/Abstract])) OR (video games[Title/Abstract])) OR (Digital health[Title/Abstract])) OR (Telehealth[Title/Abstract]))) AND ((((((((((Young Adult[MeSH Terms]) OR (college student*[Title/Abstract])) OR (university student*[Title/Abstract])) OR (Adults, Young[Title/Abstract])) OR (Adult, Young[Title/Abstract])) OR (Young Adults[Title/Abstract])) OR (undergraduate[Title/Abstract])) OR (Postgraduate[Title/Abstract])) OR (Master student*[Title/Abstract])) OR (doctoral student*[Title/Abstract])) OR (Higher education student*[Title/Abstract]))

Multimedia Appendix 1. Searching Strategy 2

Web of Science (WOS)：

#1: ((((((((((((((((((((TI=(daily physical activit*)) OR TI=(exercise)) OR TI=(walk*)) OR TI=(motor activ*)) OR TI=(leisure activit*)) OR TI=(physical fitness)) OR TI=(sport*)) OR TI=(health)) OR TI=(health behavior)) OR TI=(weight loss)) OR TI=(obesity)) OR TI=(overweight)) OR TI=(Physical activity)) OR TI=(Fitness)) OR TI=(Workout)) OR TI=(Training)) OR TI=(Athletics)) OR TI=(Recreation)) OR TI=(Behavior change)) OR TI=(Health behavior change)) OR TI=(Physical activity behavior change)

#2: (((((((((((((((((((((((((((((((((((TI=(mobile health)) OR TI=(ehealth)) OR TI=(mhealth)) OR TI=(mobile technology)) OR TI=(Internet)) OR TI=(cellular phone)) OR TI=(cellular phones)) OR TI=(smartphone)) OR TI=(telecommunications)) OR TI=(mobile applications)) OR TI=(web-based)) OR TI=(mobile apps)) OR TI=(portable electronic app)) OR TI=(portable software app)) OR TI=(text message)) OR TI=(SMS)) OR TI=(short message service)) OR TI=(portable game)) OR TI=(computers, handheld)) OR TI=(PDA)) OR TI=(personal digital assistant)) OR TI=(social media OR social media health)) OR TI=(Twitter)) OR TI=(tweeTI)) OR TI=(Facebook)) OR TI=(Instagram)) OR TI=(Wechat)) OR TI=(Tiktok)) OR TI=(mobile fitness apps)) OR TI=(online social networking)) OR TI=(virtual reality)) OR TI=(avatars)) OR TI=(online gaming)) OR TI=(video games)) OR TI=(Digital health)) OR TI=(Telehealth)

#3: ((((((((((TI=(Young Adult)) OR TI=(Adult, Young)) OR TI=(AdulTI, Young)) OR TI=(Young AdulTI)) OR TI=(university student)) OR TI=(college student)) OR TI=(undergraduate)) OR TI=(Postgraduate)) OR TI=(Master student)) OR TI=(doctoral student)) OR TI=(Higher education studenTI)

#4: ((((((TI=(clinical trial)) OR TI=(pilot study)) OR TI=(randomized controlled clinical trial)) OR TI=(Randomized controlled trial)) OR TI=(RCT)) OR TI=(Quasi-experimental study)) OR TI=(Intervention study)

Combined: #1 AND #2 AND #3 AND #4
