# Supplementary material for: Effectiveness of Digital Health Interventions in Promoting Physical Activity Among College Students: Systematic Review and Meta-Analysis
Source: J Med Internet Res. 2024 Nov 20;26:e51714. doi: 10.2196/51714 (PMC11618011; doi:10.2196/51714)
Supplement: Multimedia Appendix 2 [file jmir_v26i1e51714_app2.pdf]

Multimedia Appendix 2. Study Characteristics of selected studies.

Study (year)<br>Study design	Country	Total<br>(Intervention/Control)	Boys/girls,<br>n	Age(years),<br>Mean±SD	Analysed sample<br>(Intervention/Control)	Type of digital health intervention	Duration of intervention	Follow-up	Primary outcomes	Secondary outcomes
Hebden (2014)<br>RCT	Austraila	51(26/25)	10/41	22.8 ± 4.6	51(26/25)	**Type:**<br>Mobile health (mHealth) intervention<br>**Description:**<br>This 12-week mHealth programme addressed four key lifestyle behaviours associated with weight gain during young adulthood: physical activity and sedentary behaviour, intake of fruit and vegetables, energy-dense takeaway meals and sugar sweetened beverages(SSB).<br>**The intervention group:** received the 12-week mHealth programme in addition to the booklet and session with a dietitian. For each behaviour selected, participants received two Short Message Service (SMS) text messages and two e-mails each week of the intervention, and access to one smartphone application and one Internet forum for each of the two behaviours selected.<br>**The control group:** received the booklet and session with a dietitian.<br>**Intervention target:**<br>University students and staff aged 18–35 years	12-week	week 13	**Body mass index(BMI)**	**Physical activity:**<br>MVPA, LPA, Sedentary time, IPAQ, IPAQ(MET), Sitting time<br>Measuring tool: Actigraph accelerometer (model GT1M; Pensacola, FL, USA)<br><br>**Intake of sugar sweetened beverages(SSB)**<br><br>**Intake of energy-dense takeaway meals**<br><br>**Intake of daily intake of fruit and vegetables**
Kim (2016)<br>RCT	USA	187(101/86)	71/116	20.2 ± 1.7	187(101/86)	**Type:**<br>Wearable technology intervention combined with credit-based physical activity instructional program(PAIP)<br>**Description:**<br>The intervention group: provided with a small, lightweight activity tracker "the Misfit Flash" (Misfit Wearables Co., Burlingame, CA) combined with PAIP.<br>The control group: did not receive any additional instructions other than the scheduled class activities based on the standardized core curriculum of PAIP<br>**Intervention target:**<br>College students	15-week	(N/A)	**Physical Activity:**<br>Sedentary time, Sedentary time≥30-min, LPA, MVPA, MVPA≥30-min.<br>Measuring tool: ActiGraph Actitrainer (ActiGraph LLC, Pensacola, FL, USA)	**Other covariates:**<br>sex, college grade, race/ethnicity, height , and weight
Miragall (2018)<br>RCT	Spain	76(26/24/26)	11/65	22.2 ± 3.7	52(26//26)	**Type:**<br>Internet-based motivational intervention(IMI)<br>**Description:**<br>The self-administered IMI developed by the authors is based on the components of the Transtheoretical Model (TTM) of behaviour change. The main objective of the intervention is to deliver information to increase motivation to do PA and set individualised goals. It has a total length of 45 min, and it is delivered on a web platform.<br>The intervention group: received the access to IMI and the use of a pedometer.<br>The control group: received the use of a blinded pedometer only.<br>**Intervention target:**<br>76 sedentary or low active college students	3-week	month 3	**Average daily steps taken**<br>Measuring tool: Fitbit One pedometer.(Fitbit Inc., San Francisco)<br><br>**Physical Activity Enjoyment Scale – Short Version (sPACES)**<br><br>**Stages of Change Questionnaire for exercise (SCQ)**<br><br>**Decision Balance Questionnaire for exercise (DBQ)**<br><br>**Self-Efficacy Questionnaire (SEQ)**<br><br>**Processes of Change Questionnaire (PCQ)**	N/A
Pope (2019)<br>RCT	USA	38(19/19)	10/28	21.5 ± 3.4	38(19/19)	**Type:**<br>A combined smartwatch and theoretically based, social media-delivered health education intervention<br>**Description:**<br>Intervention group: provided a Polar M400 smartwatch to track PA duration and steps/day and included in a Facebook group wherein SCT-(social cognitive theory) and SDT-(self-determination theory) based PA and nutritious eating health education tips were provided twice weekly.<br>Control group: included only in separate, but content-identical, Facebook group, with no smartwatch provided.<br>**Intervention target:**<br>College students	12-week	(N/A)	Intervention Interest,<br>Use/Acceptability,<br>Adherence,<br>Retention.	**Physical Activity:**<br>MVPA, LPA, SB<br>Measuring tool: ActiGraph Link accelerometers<br><br>**Cardiorespiratory Fitness:**<br>beats-per-minute<br><br>**Height, weight, and body composition**<br><br>**Psychosocial Variables:**<br>Self-Efficacy, Social Support, Enjoyment, Barriers, Outcome Expectancy, Intrinsic Motivation<br><br>**Dietary Behaviors:**<br>Daily Caloric Consumption, Daily Fruit Intake, Daily Vegetable Intake, Daily Whole Grain Intake, Daily Sugar-Sweetened Beverage Kcalories
Al-Nawaiseh (2022)<br>RCT	USA	114(56/58)	22/92	21.1 ± 2.0	114(56/58)	**Type:**<br>M-Health applications intervention<br>**Description:**<br>This test Used one of the most popular publicly available Smartphone apps for improving the PA (Step counts) (Pacer). It has goal setting functionality, self-monitoring of step counts, calories expended, and automatic performance feedback through the graphic display of step-count history.<br>The intervention group received PA goals in terms of 10,000 steps/day and received information about the benefits of exercise and instructions on how to use the app. By the end of each week (week 2 to week 12), each participant in the intervention group was contacted via SMS/e-mail and asked to share their step count data with the researcher.<br>The control group were provided with information related to daily recommended PA levels and information highlighting the benefits of walking regularly, without being observed or requiring interaction with the	12-week	week 12	**Physical activity:**<br>Step counts<br>Measuring tool: (Pacer) pedometer m-Health based-app	**Anthropometric Measurements:**<br>Body weight, Fat%, and BMI
Lau (2022)<br>RCT	China	56(28/28)	19/30	21.5 ± 1.7	49(24/25)	**Type:**<br>4-week Facebook PA Intervention<br>**Description:**<br>The intervention group: received education links and intervention messages on the Facebook page posted by the captains, and were required to complete a 14-item questionnaire on the page each day.<br>The Control group: received no such intervention messages and had no group captain or discussion; they only finished the questionnaires.<br>**Intervention target:**<br>College students aged 18-25	4-week	(N/A)	**Objective PA measure:**<br>MVPA, MVPA(%),Average vector magnitude of all 3 Axis, step counts<br><br>Measuring tool:<br>An activity monitor from ActiGraph (Pensacola, FL) for MVPA, MVPA(%), Average vector magnitude of all 3 Axis.<br>A free version mobile App named Accupedo pedometer for Setp counts.	**Daily physical activity-related information:**<br>commuting type, sport type, sport venue, sport duration, and intensity.<br><br>**Psychological aspects of PA:**<br>perceived PA level, stage of readiness in PA participation, effectiveness and efficiency, and attractive features
Pope (2022)<br>RCT	USA	44(22/22)	12/32	21.6 ± (n/a)	44(22/22)	**Type:**<br>A smartphone application and theoretically-based, social media-delivered health education intervention<br>**Description:**<br>The intervention group: used "MapMyFitness" smartphone application to log and track physical activity and participated in a Social Cognitive Theory-based(SCT), Facebook-delivered health education intervention,<br>The control group: only included in a separate, but content-identical, Facebook intervention.<br>**Intervention target:**<br>College students	10-week	(N/A)	Intervention interest,<br>use/acceptability,<br>Retention,<br>Feasibility.	**Physical activity levels:**<br>MVPA, SB<br>Measuring tool: Actigraph GT3X accelerometers<br><br>**Anthropometry, body composition, and cardiovascular fitness.**<br><br>**Psychosocial variables:**<br>Self-Efficacy, Social Support, Enjoyment, Barriers, Outcome Expectancy
Kellner (2023)<br>RCT	German	34(16/18)	5/29	22.3 ± 2.6	34(16/18)	**Type:**<br>Messenger-based Intervention<br>**Description:**<br>The intervention group: received two messages daily (in the morning and in the evening) which contained prompts to interrupt sitting time and suggestions for isotemporal substitution of sedentary behavior.<br>The Control group: was designed as a waiting-control group and received the messages for 3 weeks after the post measurement.<br>**Intervention target:**<br>College students	5-week	(N/A)	**Sedentary time**<br>Measuring tool: ActivPal accelerometers (Pal Technologies Ltd., Glasgow)	**Evaluation of the messages: (six questions)**<br>feasibility of the tasks,<br>consequent adherence to tasks,<br>if the tasks were fun,<br>if the frequency of the messages was appropriate,<br>if the lengths of the messages was appropriate,<br>if the tasks were executed even after the end of the intervention.
